# Migraine and Epilepsy*Editorial in Journal American Medical Association, April 9, 1898.

**Published:** 1898-05

**Authors:** 


### MIGRAINE AND EPILEPSY.*

The pathologic etiology of migraine has been long enough the subject of speculation, but we are still in the phase of conjecture or at least theory; our positive acquisitions of knowledge as to the essential pathologic process are so far practically nothing. The notion that it is a vasomotor neurosis has been largely abandoned, and neurologists at the present time are inclined to class it with epilepsy as a fulgurant cortical neurosis, a symptom of cortical instability associated, it may be, with some conditions of morbid metabolism, such as the uric acid or arthritic diathesis. While it may be assumed that it is a derangement to some extent of the function of the sensory cortical elements due to various irritations arising from the digestive organs, the sexual apparatus, the general or spinal sensory tracts, etc., its ultimate cause must be looked for somewhere back of all these in some more profound systemic condition, the exact nature of which we are only able to conjecture at the present time.

Dr. B. K. Rachford, who has, during the past few years, advocated the theory of leucomaine poison as the essential cause of this disorder, publishes in the April issue of the *American Journal of Medical Sciences* a further paper on the subject, reiterating his former views and supporting them by additional arguments and facts. His former findings of xanthin and paraxanthin in the urine of sufferers from migraine have, he states, been confirmed by continued observations during the past two years, especially as regards the latter. In normal urine paraxanthin is present in too minute a quantity to be readily detected, and this is true also of that secreted in ordinary attacks of headache, which have not the clinical features of what we recognize as migraine. He does not, therefore, believe that cephalalgia is itself necessarily or directly associated with this excess of paraxanthin; it is the special condition that gives rise to the peculiar migrainous attack. An over-production and accumulation of this product in the blood from some recondite neurotic condition appears to be his theory, and the migrainous explosion is the attendant of its periodical excretion. It is the most toxic of all the leucomaines of the uric acid group, and some of the symptoms it produces are suggestive of those of migraine. From all the data thus far obtained, Dr. Rachford deems it reasonable to assume that paraxanthin is to be considered as an important factor, at least in the production of true migraine.

The relations of epilepsy and migraine suggested in the theory stated as just now prevalent, are quite strongly suggested by certain clinical facts familiar to neurologists and to whoever has any extensive experience with these neuroses.

*Editorial in Journal American Medical Association, April 9, 1898.

Epileptiform migraine with loss of consciousness, more or less complete, in some of the attacks, and even with convulsive manifestations, is a well-known condition, and the disorder has unquestionably a close relation with the more formidable affection, as do also certain forms of vertiginous attacks, themselves apparently related to migraine. The pathology of epilepsy, considered as a disease of itself, is also obscure, but when it is reckoned only as a symptom of various pathologic states it is possibly more comprehensible, or at least more easily explained in a theoretic way. Dr. Rachford makes this point in his paper and indicates the distinctions between organic or mechanical, reflex and toxic epilepsies, the first being readily comprehensible, the second due to lack of inhibition from defective development due to inheritance or malnutrition, and the last as yet imperfectly understood, but probably not the least in importance of the three general types. As a special form of toxic epilepsy he recognizes that from paraxanthin intoxication, which, according to his investigations, forms a small but appreciable proportion of the whole class, and has some special features of its own, though hardly enough to characterize it clinically as a definitely marked type. It is more common in women than in men, in middle than in early life, and always in cases that have suffered from, or are still subject to, migraine, thus indicating clearly the common causal factor. It is compatible with unimpaired intellectual powers, thus differing from most of the other forms of epilepsy, and is generally more amenable to treatment, thus rendering an early diagnosis the more important.

It would appear from the facts that Rachford has collected, that there is a common cause in certain cases of epilepsy and of migraine, and that, as he is inclined to believe, this is to be found in the toxic action of certain leucomaines of which paraxanthin is probably the most important. That these do not invariably cause epilepsy or migraine may be admitted, but that is one of the very common facts for which we can have as yet no explanation, and which does not vitiate the conclusion that there is sometimes a causal relation between their occurrence and that of these disorders.

There are undoubtedly other autotoxic factors in epilepsy, those, for example, from the intestinal organs, and the analogy of drug epilepsies is sufficient to make this probable. It is an interesting fact none the less that we can in this way find a plausible explanation of the long recognized affinities of migraine and epilepsy, and the line of investigation here indicated is well worthy of being further followed out. It may throw light not only on the causes but also on the essential lesion, and the practical questions of treatment and prophylaxis.—*American Therapist.*

